# No distance is too far between friends: Associations of comfortable interpersonal distance with PTSD and anxiety symptoms in traumatized individuals

**DOI:** 10.1192/j.eurpsy.2021.1199

**Published:** 2021-08-13

**Authors:** S. Haim-Nachum, M.R. Sopp, T. Michael, S. Shamay-Tsoory, E. Levy-Gigi

**Affiliations:** 1 Education, Bar-Ilan University, Ramat Gan, Israel; 2 Department Of Psychology, Saarland University, Saarbrücken, Germany; 3 Psychology Department, University of Haifa, Haifa, Israel

**Keywords:** ptsd, Anxiety, ERP, Interpersonal distance

## Abstract

**Introduction:**

Previous research indicates that traumatized individuals with post-traumatic stress disorder (PTSD) symptoms may show alterations in interpersonal distance regulation that are not evident in traumatized individuals without PTSD symptoms. However, the underlying mechanisms of these alterations are yet to be investigated. Moreover, it is not clear whether altered interpersonal distance regulation is correlated with trauma-related psychopathology.

**Objectives:**

The current study investigated behavioral and neurophysiological markers of interpersonal distance regulation as predictors of PTSD and anxiety in traumatized firefighters.

**Methods:**

Twenty-four active-duty firefighters (M = 30.58, SD = 3.62) completed an experimental task that measures comfortable interpersonal distance. During the task, event-related potentials were recorded to assess attentional processing as reflected in the P1 and N1 components. Trauma-related psychopathology was assessed using the Clinician-Administered PTSD Scale and the state version of the State-Trait Anxiety Inventory.

**Results:**

Participants who did not choose a closer distance towards friends as compared to strangers experienced greater anxiety post-trauma. On a neurophysiological level, participants who showed attentional avoidance towards strangers reported more PTSD symptoms. By contrast, participants who showed hypervigilant attention towards strangers reported greater anxiety.

**Conclusions:**

The results demonstrate associations between interpersonal distance regulation and psychopathology after trauma, shedding light on the underlying processes of interpersonal distance regulation in anxiety and PTSD. Future studies should re-investigate these associations in a larger sample and explore potential implications for the prevention and treatment of trauma-related psychopathology.

